# Investigation of a non-invasive method of assessing the equine circadian clock using hair follicle cells

**DOI:** 10.1186/1740-3391-10-7

**Published:** 2012-10-05

**Authors:** Lisa M Watts, John A Browne, Barbara A Murphy

**Affiliations:** 1 School of Agriculture and Food Science, University College Dublin, Belfield, Dublin 4, Ireland

**Keywords:** Equine, Horse, Clock, qPCR, Hair follicle, PER1, PER2, DBP, NR1D1, Circadian

## Abstract

**Background:**

A comprehensive understanding of the equine circadian clock involves the evaluation of circadian clock gene expression. A non-invasive and effective method for detecting equine clock gene expression has yet to be established. Currently, research surrounding this area has relied on collecting tissue biopsies or blood samples that can often be costly, time consuming and uncomfortable for the animal.

**Methods:**

Five mares were individually stabled under a light–dark (LD) cycle that mimicked the external environmental photoperiod during a time of year corresponding with the vernal equinox. Hair follicles were collected every 4 h over a 24-h period by plucking hairs from the mane. RNA was extracted and quantitative (q) PCR assays were performed to determine temporal expression patterns for the core clock genes; *ARNTL, CRY1, PER1, PER2, NR1D2* and the clock controlled gene, *DBP*.

**Results:**

Repeated measures ANOVA for the clock gene transcripts *PER1* and *PER2* and the clock controlled gene, *DBP*, revealed significant variation in expression over time (*p < .05*, respectively). Cosinor analysis confirmed a significant 24-h temporal component for *PER1* (*p = .002*) and *DBP (p = .0033)* and also detected rhythmicity for *NR1D2 (p = .0331)*.

**Conclusions:**

We show that the extraction of RNA from equine hair follicle cells can identify the circadian 24 h oscillations of specific clock genes and a clock-controlled gene and therefore provide a valuable non-invasive method for evaluating the equine peripheral circadian clock. This method will serve as a useful tool for future evaluations of equine circadian rhythms and their response to environmental changes.

## Background

The circadian system supplies organisms with a means to adapt their internal physiology to the continuously changing environmental stimuli that exist on a rotating planet [1]. The central pacemaker is located in the suprachiasmatic nucleus (SCN) of the hypothalamus and coordinates, via neural and humoral signals, multiple peripheral clocks situated in tissues throughout the animal [2]. These peripheral clocks consist of a group of highly conserved ‘clock’ genes and their protein products functioning within tightly controlled autoregulatory transcription-translation feedback loops [3] that ultimately give rise to 24-h alterations in gene expression and behavioural outputs.

Circadian clock gene expression is detectable not only in the SCN but in almost all peripheral tissues. The molecular clockwork mechanism comprises a series of feedback loops that encompass a set of core clock genes: *ARNTL* (aryl hydrocarbon receptor nuclear translocator-like), *CLOCK* (circadian locomotor output control kaput), *PER1* (period homolog 1), *PER2* (period homolog 2), *PER3* (period homolog 3), *CRY1* [cryptochrome 1 (photolyase-like)], and *CRY2* [cryptochrome 2 (photolyase-like)] [4].

The positive axis of the loop is created by transcription factors *CLOCK* and *ARNTL* as they undergo transcription and translation [3]. *CLOCK* and *ARNTL* proteins heterodimerize and bind to E-box enhancers upstream of *PER* and *CRY* genes in order to trigger transcription [5]. Following this, a complex is formed by *PER* and *CRY* proteins that relocates to the nucleus to inhibit *CLOCK/ARNTL* activity. This leads to the repression of their own transcription completing the negative axis of the feedback loop [2]. *RORA*, (RAR-related orphan receptor A) *NR1D1* (nuclear receptor subfamily 1, group D, member 1), and *NR1D2* (nuclear receptor subfamily 1, group D, member 2) are orphan nuclear receptors that make up a secondary feedback loop. *RORA* instigates *ARNTL* transcription whilst *NR1D1* and *NR1D2* repress its expression [6]. Each cycle of the molecular clock within a tissue gives rise to the simultaneous upregulation of a subset of clock-controlled genes [7] activated by the transcriptional activity of the ARNTL/CLOCK heterodimer.

Markers of circadian phase in humans include melatonin [8] and body temperature [9]; however melatonin has been established as not circadian in the horse [10]. Previous studies in humans have used white blood cells or oral mucosa as a method of detecting human clock gene expression [11,12]. These methods have several reported drawbacks [11,13]. Physical stimuli and time delays due to the processing of cell separation may affect levels of expression of clock genes and the overall quality of the isolated mRNA. For instance with white blood cells, the issue relates to time delays due to the processing of cells prior to transcriptional inactivation which in turn may affect the levels of mRNA of clock genes. A similar concern can be seen with the collection of oral mucosa cells. RNA samples were shown to be severely fragmented and thus the results were discarded [12]. There are additional impracticalities in collecting oral mucosal cells from horses. These could be avoided through the collection of hair follicles as an alternative sampling method [13]. The main advantages of using hair follicle cells are that they can be obtained noninvasively and cells can be collected and the RNA stabilised simply by plucking hairs and adding them directly to an RNA stabilisation buffer.

A non-invasive and effective method for detecting peripheral equine clock gene expression has yet to be established. This hinders progress in areas of equine circadian research. Currently, research investigating equine peripheral clocks has relied on collecting muscle biopsies or blood samples. These methods can often be time consuming, costly and can occasionally lead to animal welfare concerns as this approach to collecting samples can be invasive.

In this report we examine a convenient and non invasive method for detecting equine clock gene expression through the use of hair follicle cells collected from the mane of the horse.

## Materials and methods

### Sample collection

Five healthy, non-pregnant mares (*Equus caballus*) of various lightweight breeds were individually housed in standard 12 ft x 12 ft stalls for 24-h under a light–dark (LD) cycle that mimicked the environmental photoperiod for that time of year. Mares were chosen for their availability on UCD’s Lyons Research Farm. Stallions were not available for the study and castrated males (geldings) are considered unsuitable as their neuroendocrine system is compromised. The experiment was conducted in March where the times of dawn and dusk were 06:00 and 18:00 respectively, corresponding to a 12 h Light : 12 h Dark LD cycle at longitude 138 W6.8, latitude N53.2 (County Kildare, Ireland). While stabled, horses had access to hay and water *ad libitum.* We collected mane hair samples at 4-h intervals from 16:00 [Zeitgeber Time (ZT) 9, where time of lights on defines ZT 0] for a period of 24 h.

Each hair sample consisted of 10–20 hair follicles that were trimmed to remove excess hair and carefully placed in a 2 mL screw-cap tube containing 400 ul of binding buffer from the High Pure RNA Isolation Kit (Roche, Indianapolis, Indiana). 

### Quantitative polymerase chain reaction (qPCR)

Total RNA was isolated using the High Pure RNA Isolation Kit (Roche, Indianapolis, INdiana) according to the manufacturer's instructions with minor modification: The hair follicles were placed in a 2.0 mL screw cap tube, containing 400 uL of binding buffer from the High Pure RNA Isolation Kit (Roche) and 200 uL of PBS. A single 5 mm stainless steel bead (Qiagen) was added to each tube and the samples were homogenised at maximum speed (30 Hertz) for 2 min using the Qiagen TissueLyser system. Following homogenisation the samples were spun for 1 min at maximum speed to reduce foaming, the homogenate was then applied to the filter column and RNA was extracted as per instructions for the High Pure Isolation Kit. RNA was eluted in 50 uL and stored at -80 deg C.

RNA quantity was measured using the NanoDrop ND1000 spectrophotometer V 3.5.2 (NanoDrop Technologies, Wilmington, DE). RNA quality was assessed using the Agilent Bioanalyser RNA Chip (Santa Clara, California). All samples were shown to have a RIN value in excess of 7.5. The RNA was converted to complementary (c) DNA and a cDNA pool containing 3.5 ul from each sample was prepared and used to generate a 7 point, 1 in 4 serial dilution. This serial dilution was used to test the efficiency of each primer pair used in the study. The remaining cDNA was diluted to 2.0 ng/ul of RNA equivalents and stored at −20°C. A number of minus reverse transcription (RT) controls were included during the cDNA preparation.

Quantitative PCR assays were performed using Biosystems 7500 Sequence Detection System and the Sensi Mix SYBR Kit (Bioline, Taunton, Massachusetts). A panel of eight putative reference genes was assessed for stability using the GeNorm algorithm with the qBase Pair Softwear package. Results showed that *ARTB* and *HPRT* were suitable reference genes and the optimal normalization factor was calculated as the geometric mean of these two reference targets. Each PCR reaction was prepared in duplicate and in a volume of 20ul [10 ul master mix, 5.0 ul cDNA, 1.2 ul forward (300 uM), 1.2 ul reverse (300 uM) and 2.6 ul water]. A panel of five core clock genes was selected; *PER1* (period homolog 1), *PER2* (period homolog 2), *ARNTL* (aryl hydrocarbon receptor nuclear translocator-like), *CRY1* [cryptochrome 1 (photolyase-like)], *NR1D2* (nuclear receptor subfamily 1, group D, member 2) and the clock controlled gene (CCG) *DBP* (D-site of albumin promoter binding protein). Candidate genes were selected based on prior evidence of their cyclic expression in human hair follicles [13] and where equine primer sequences were previously published [14]. Primers sequences were commercially synthesised by Eurofins MWG Operon (Ebersberg, Germany).

Thermal cycling consisted of one cycle of 50°C for 2 min and 95°C for 10 min, followed by 40 cycles at 95°C for 15 seconds and 60°C for 1 minute. Melt curves were examined to confirm specificity of each PCR product. Primer efficiencies were shown to be between 90% and 110%. Transcript abundance was determined relative to *ACTB* and *HPRT1* using the Q Base Plus Software package (Biogazelle, Belgium). CNQR results were analysed using SPSS.

### Data analysis

One-way repeated measures ANOVA (GraphPad Prism Version 5.0 for Mac, GraphPad software, San Diego, California, USA, http://www.graphpad.com) was used to determine whether the temporal pattern of expression for each transcript varied significantly over the 24-h period. The presence of diurnal (24-h) temporal variation for transcript means was evaluated using a Cosinor programme [15] based on the least squares cosine fit method [16]. In all cases significance was assessed as *p < .05*. Data are presented as means ± SEM.

## Results

**Figure 1:**
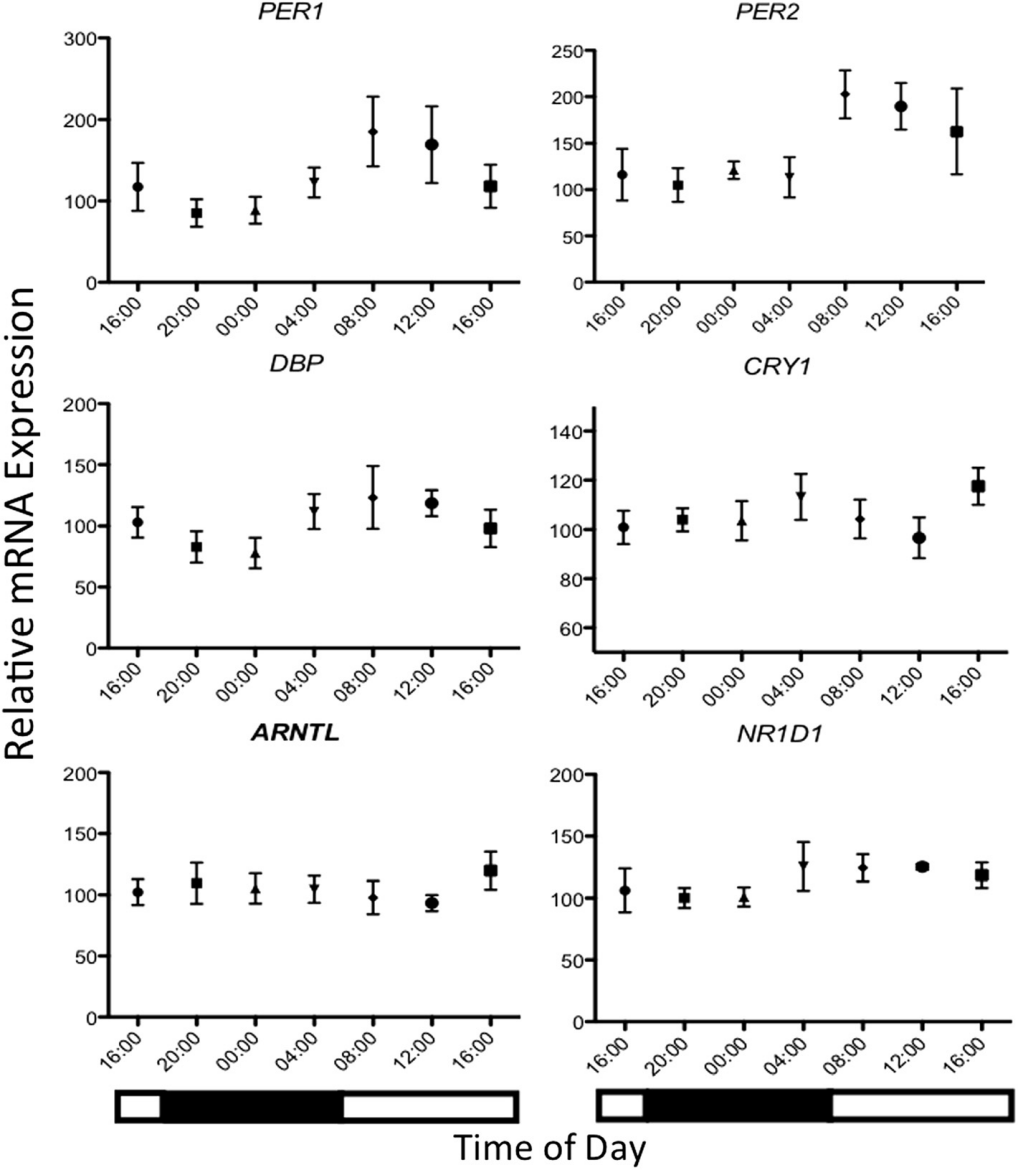
**24-h profiles of mRNA expression.** Plotted are mRNA levels of the candidate genes relative to reference genes *ACTB* and *HPRT1* in equine hair follicles. Data are presented as means ± SEM. Black and white bars indicate hours of darkness and light, respectively. ANOVA revealed significant 24-h variation for *PER1, PER2* and *DBP (p = .024, p = .02* and *p = .03;,* respectively) whereas Cosinor analysis confirmed a significant 24-h component for *PER1, DBP* and *NR1D2 (p = .002, p = .0331 and p=.03;,* respectively.

The expression patterns of five core clock genes *PER1, PER2, ARNTL, CRY1* and *NR1D2* and the clock controlled gene, *DBP* (D-site of albumin promoter binding protein) were investigated in this study. We detected mRNA expression of all six genes in equine hair follicles. ANOVA revealed significant 24-h variation for three of the genes *PER1, PER2* and *DBP (p = .024, p = .02* and *p = .036,* respectively*;* Figure 1) but not for the remaining three genes *ARNTL, CRY1* and *NR1D2 (p = .58, p = .32, p = .37,* respectively; Figure 1).

Cosinor analysis confirmed a significant 24-h cyclic component for *PER1 (p = .002), DBP (p = .0033)* and *NR1D2 (p = .0331)* while the 24-h cosine fit for *PER2* (p = .0643) was just shy of significance. As the inverse oscillatory relationship between *PER2* and *ARNTL* expression is considered a characteristic component of the molecular clockwork mechanism, we further examined whether any individual expression profiles of *ARNTL* exhibited a circadian waveform. No individual 24-h expression profiles for *ARNTL* exhibited significance via cosinor analysis (*p* > .05) Results of cosinor analysis including estimated acrophases are presented in Table 1.

**Table 1:** Results of cosinor analyses of mRNA expression profiles

**Gene transcript**	**Robustness (%)**	**Acrophase (24 h)**	**Zeitgeber Time (ZT)**	***p*value**
*PER1*	94	10:33	4.5	*0.002*
*DBP*	92	08:37	2.5	*0.0033*
*NR1D2*	65	08:57	3.0	*0.0331*
*PER2*	51	09:33	3.5	*0.0643*
*CRY1*	0	n/a	n/a	*0.8944*
*ARNTL*	0	n/a	n/a	*0.3066*

For each gene transcript the robustness values, acrophase time, corresponding Zeitgeber Time and and significance values (p) are presented.

## Discussion

In this study, we demonstrate that RNA extraction from equine hair follicle cells yields high quality RNA and is suitable for detection of 24 h oscillations of core components of the equine molecular clock. To demonstrate that oscillating clock gene expression in hair follicle cells can be used as markers to assess an equine peripheral clock, we examined gene expression in hair follicle cells collected from the manes of five mares over a 24 h period. This is the first study to investigate a non-invasive method of assessing an equine peripheral tissue clock.

We show that mRNA levels of *PER1, PER2* and *DBP* vary significantly over time as determined by ANOVA. However, of these three, only *PER1* and *DBP* are shown to exhibit a 24-h sinusoidal profile as determined by cosinor analysis. Furthermore, although non-significant by ANOVA, the expression profile of *NR1D2* is positive for 24-h rhythmicity by cosinor analysis. These apparent discrepencies can be explained by understanding the nature of each analysis. Cosinor analysis is more sensitive than ANOVA at picking up sinusoidal patterns in the data associated with a specified period as that is precisely what it is designed to do. The ANOVA can take into account the repeated measures design in partitioning out the variance but has no means of accounting for the temporal relationship (angular proximity) of the time points and no model for temporal variation against which the observed variation is compared. In the case of *PER2,* the highly variable means at specific time points were detected by ANOVA but the angular proximity of the time points were only weakly sinusoidal. Conversely, while the mean expression of *NR1D2* at specific time points varied to a lesser extent, the angular proximity of the data points more closely reflected a sinusoidal pattern. Of the six gene transcripts examined, we find that *PER1* and *DBP* may serve as the most reliable markers for this peripheral clock in the horse with limited sampling frequency.

Furthermore, we report that as few as 10 equine hairs are sufficient for high quality RNA yield. A significant advantage of this technique is that it can be carried out by an untrained person and eliminates the need for tissue biopsies.

A comparison of our results with a previous study evaluating clock gene expression from human hair plucked from the scalp and chin [13] revealed that a similar temporal pattern of expression of *PER2, NR1D2* and *DBP* exists between species when maintained under LD12:12. Although the LD cycle under which the human subjects were maintained is not reported, the three oscillating genes in common to the two studies, *PER1, DBP* and *NR1D2*, exhibit peaks in the morning between 07:00 and 09:30, suggesting a similar phase relationship between these genes in the two species.

Importantly, in a previous investigation of clock gene expression profiles in equine gluteal muscle conducted by our lab [14], peak values of *PER2, NR1D2* and *DBP* were observed at 07:00. Although further studies are required to accurately identify the phase relationship between the peripheral clocks in muscle tissue and hair follicles, these findings suggest that hair follicle expression profiles may provide a valuable marker with a similar phase relationship to the envitronmental LD cycle as a more performance relevant equine tissue, gluteal muscle.

It was surprising to fail to find rhythmic expression of the core clock genes, *ARNTL* and *CRY1*, in hair follicle cells. However, it has become clear from studies in peripheral tissues that the specific contributions and interactions between specific clock components vary in a tissue-specific manner [4]. Moreover, in the recent human study of hair follicle clock gene expression, only “slight oscillations” of *ARNTL* were detected while *CRY1* was not examined [13]. It cannot be excluded that the cycling amplitude of *ARNTL* and *CRY1* in follicle cells may be inherently small and undetectable in the current assays, or that post-translational processing plays a more pivotal role in this tissue clock.

Circadian rhythms regulate hundreds of functions relevant to the athletic horse including body temperature and hormone production [10,17], immune function [18] and muscle metabolism [14]. Disruption of the circadian system could have a profound influence on equine health. In humans, the disruption of circadian rhythms has been linked to jetlag [19], insomnia [20], stomach ailments [21] and depression [22]. With more research into the characterisation of the circadian clock, it may be possible to pin point the exact consequences of disruption to the circadian cycle on the equine system.

The need for a non-invasive marker of circadian phase is particularly relevant for furthering equine research in relation to transmeridian transportation of horses for international competition. It is well accepted that the circadian clock regulates activity and also muscle metabolism in mammals [23,24]. This is supported by evidence that nocturnally active rats experience peak expression of *PER2* in skeletal muscle at the onset of dark [23,24] whereas diurnally active horses have an opposing peak of *PER2* expression at dawn [14]. The capacity of the molecular clock within the SCN to reset following an abrupt 6-h LD shift, as occurs during transmerdian travel across six time zones, was shown to take up to eigth days in mice [25]. The gradual re-entrainment of clock genes within specific areas of the SCN was correlated with, and mirrored, a disruption in circadian behavioral output, as measured by locomotor activity rhythms in a further study [26].

In addition to desynchronized clock gene expression within the master pacemaker in the SCN, rhythmic gene expression in peripheral tissues, which rely on SCN signals for synchrony, are also significantly disrupted. This was clearly demonstrated in rats when it was reported that clock gene cyclicity in skeletal muscle, liver and lung shifted more slowly than the SCN following both LD cycle advances and delays [27]. The authors concluded that this likely further explained the physical malaise in humans associated with rapid transmeridian travel.

The capacity to evaluate markers of circadian phase, as determined by clock gene expression patterns, to determine the extent and duration of circadian misalignment in response to abrupt changes in the 24-h LD cycle, as occurs during transmeridian travel, represents a valuable experimental tool. Our results suggest that equine hair follicles may be used in future studies as a reliable and non-invasive method to detect the time duration required for a peripheral equine tissue to adjust to a new time zone. If it is found that the phase of the equine hair follicle clock closely mirrors that in skeletal muscle, which our results suggest, and exhibit similar re-entrainment rates following simulated jet lag, then there clearly exisits the opportunity to quantify the duration of potential performance deficits and to develop a molecular test for time zone resynchronization in these valuable global athletes. This in turn could help horse trainers determine how much time in advance to transport their racehorse to a temporary location before an important race.

## Conclusions

We demonstrate that RNA extraction from equine hair follicle cells is a suitable method of evaluating certain core clock genes and a clock-controlled gene to assess an equine peripheral clock. In particular, our findings support the evaluation of the gene transcripts *PER1* and *DBP* in hair follicle cells as suitable markers for evaluating the phase of a peripheral clock in the horse.

## Competing interests

The authors declare that they have no competing interests.

## Authors’ contributions

LMW collected the data, performed the gene expression assays, analysed data and drafted the manuscript. BAM conceived of the project, assisted with data collection and analysis and drafted the manuscript. JAB supervised gene expression assays and assisted with drafting manuscript. All authors approved the final manuscript.
